# Multiple myeloma presenting as a massive osteolytic lesion at the clivus

**DOI:** 10.1002/jha2.106

**Published:** 2020-09-18

**Authors:** Domingos Sousa, Maria Eduarda Couto, Alda Tavares, Nelson Domingues, Isabel Oliveira, Mário Mariz

**Affiliations:** ^1^ Internal Medicine Department Centro Hospitalar e Universitário do Algarve, E.P.E. Faro Portugal; ^2^ Onco‐hematology Department Instituto Português de Oncologia do Porto, F.G., E.P.E. Porto Portugal; ^3^ Medical Oncology Department Hospital Pedro Hispano Matosinhos Local Health Unit Matosinhos Portugal

A 63‐year‐old man presented to the emergency department with acute confusion and a 1‐month history of frequent falls. Neurological examination revealed anisocoria, unresponsiveness to light stimulation and ophthalmoplegia in the right eye. Brain computed tomography (CT) demonstrated a mass at the cranial base (Figure 1). Additionally, brain magnetic resonance imaging (MRI) revealed an extensive infiltrative lesion, centered on the clivus (Figure 2). A biopsy of the left nasal fossa showed a neoplasm consisting of relatively small cells, with immunochemistry positivity for vimentin, CD56 and CD138. The serum protein electrophoresis showed an M peak of 12.6 g/dL. Whole‐body X‐ray studies revealed multiple lytic cranial lesions. The bone marrow aspirate showed 10‐15% plasmacytosis, kappa restricted.

**FIGURE 1:**
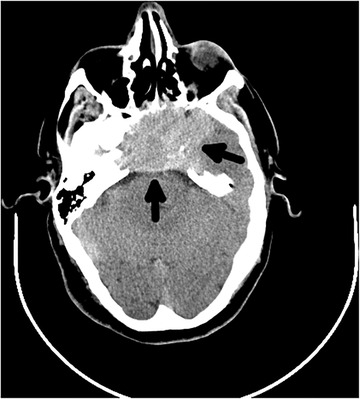
Axial plane of brain CT with a massive infiltrative lesion with 63 mm

**FIGURE 2:**
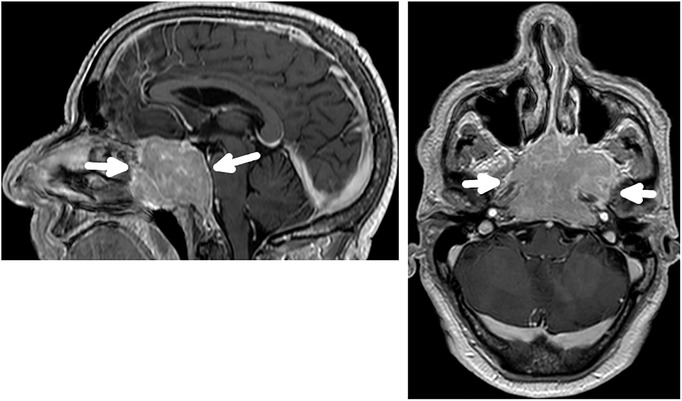
T2‐weighted brain MRI shows an infiltrative lesion centered on the clivus with contrast enhancement invading both cavernous sinuses on sagittal and axial views

This is a rare presentation of multiple myeloma (MM) with a massive osteolytic lesion at the clivus. Given its availability, brain CT is usually the first imaging modality; however, MRI is fundamental for a better characterization of the MM lesions at the skull base that are typically isointense or hypointense on T1‐weighted MRI, but hyperintense on T2 [1, 2, 3, 4]. Other differential diagnosis include plasmacytoma, chordoma, osteosarcoma, nasopharyngeal carcinoma, meningioma, metastatic carcinoma, and lymphoma [1, 2, 3].

An accurate diagnosis is extremely important for skull base masses as treatment and prognosis differ. MM with soft tissue masses has an unfavorable prognosis and the medical approach depends on the patient's functional reserve and available therapeutic arsenal [2, 3, 4].

## CONFLICT OF INTEREST

The authors declare no conflict of interest.

## HUMAN AND ANIMAL RIGHTS

This article does not contain any study with human and animals performed by any of the authors.

## INFORMED CONSENT

Informed consent was signed.

## AUTHOR CONTRIBUTIONS

Acquisition of data, clinical and imaging data review, literature review, and final manuscript writing: Sousa. Important intellectual contribution and final manuscript writing: Couto, Tavares, Domingues, Oliveira, and Mariz.
